# Influence of Cr and Cd accumulation and excretion on growth and gut microbiota in black soldier fly larvae (Diptera: Stratiomyidae)

**DOI:** 10.3389/fvets.2026.1777366

**Published:** 2026-04-15

**Authors:** Jianwei Hao, Huilin Lang, Shuang Liu

**Affiliations:** 1 Department of Biological Science and Technology, Jinzhong University, Jinzhong, China; 2 Shanxi Key Laboratory for Ecological Restoration of Loess Plateau, Shanxi University, Taiyuan, China

**Keywords:** frass, heavy metal, *Hermetia illucens*, larval development, larval gut, microbial community

## Abstract

Black soldier fly larvae (BSFL) are widely used in livestock manure treatment; however, heavy metal contamination in manure poses risks to their growth and application, limiting their practical promotion. However, existing studies have predominantly focused on the effects of individual heavy metals on BSFL, whereas the combined effects of Cr and Cd remain unclear. Furthermore, the underlying mechanisms by which Cr and Cd affect BSFL growth through gut microbiota regulation remain poorly understood. This study aimed to evaluate the effects of Cr and Cd accumulation and excretion on larval growth and gut microbiota and to clarify the related regulatory mechanisms. BSFL were reared on feed mixtures with different ratios of Cd and Cr exposure. The Cr and Cd contents in larval bodies and frass, as well as larval growth performance, behavior, nutrient composition, gut damage, and gut microbial community structure, were measured and analyzed. The results showed that Cr and Cd accumulation in larval bodies and frass increased significantly with increasing exposure concentration, and Cd was more readily accumulated (60.4–86.8%) than Cr (<15%). Co-exposure to Cr and Cd inhibited larval development, reduced larval weight, increased crude protein (CP) content, decreased ether extract (EE) content, and caused significant gut damage. Additionally, the diversity and complexity of the larval gut microbial community increased significantly (*p* < 0.05, LSD test). Proteobacteria and Firmicutes were positively correlated with larval weight and EE, while Bacteroidetes and Actinobacteria were negatively correlated with these parameters, suggesting that Cr and Cd accumulation and excretion affect larval growth by altering the gut microbiota. This study contributes to the theoretical framework of heavy metal–gut microbiota–host interactions in BSFL, addresses the research gap regarding the combined effects of Cr and Cd on BSFL, and provides a scientific basis to guide the safe and efficient application of BSFL in the treatment of heavy metal-contaminated livestock manure, thereby supporting its important academic and practical relevance.

## Introduction

The growing demand for meat, milk, and other animal byproducts is expected to increase the volume of livestock waste (1, 2). Livestock manure is rich in nutrients, but it is also a substantial source of organic pollutants, heavy metals, and disease-causing agents (3, 4). Currently, only 20–30% of animal and poultry manure is processed through traditional methods, including composting, co-composting, and anaerobic digestion (5). The treated manure is then used as a soil organic fertilizer or to produce methane for energy (2, 6). However, because of the high initial investment costs and limited land availability, these conventional methods are labor-intensive, generate low-quality commodities, and are time-consuming (7). Furthermore, both unprocessed and conventionally treated livestock manure can cause serious environmental issues, including hazards to air, soil, and water quality and the spread of pathogens (8, 9).

More efficient handling and management of animal manure are urgently needed. Thus, the use of insect bioconversion to treat excess manure and recycle nutrients in the food chain has increased (10). The larvae of the black soldier fly, *Hermetia illucens* (L.) (Diptera: Stratiomyidae), can consume a wide range of decomposing organic waste materials, including plant material, animal manure, and food and kitchen waste. These materials are transformed into organic fertilizers with antimicrobial peptide activities (11, 12), which can inhibit pathogen growth and reduce pathogen levels and heavy metals in their growth substrates (4, 13, 14). As a result, black soldier fly larvae are considered a promising tool for managing animal manure in the future (10, 15).

Some insect species used as animal feed have been found to contain heavy metals, which pose safety risks (16). A typical example is black soldier fly larvae, which are widely used as decomposers to treat organic waste and as feed for poultry and livestock. These larvae can absorb and accumulate heavy metals during waste decomposition (17); therefore, larvae used in composting and manure treatment may also pose heavy metal-related risks when used as animal feed (3). Previous studies have demonstrated that the accumulation patterns of heavy metals in black soldier fly larvae differ significantly depending on the metal type and exposure concentration (17–21). For example, Gao et al. (20) reported that a significant difference was observed in the bioaccumulation factor of black soldier fly larvae for Cd and Cr, with Cd exhibiting a stronger bioaccumulation capacity, whereas Cr showed a lower capacity. Similarly, Wu et al. (22) demonstrated that Cd exhibited a significantly higher bioaccumulation potential in BSFL bodies than Cr. However, few studies have investigated how heavy metal accumulation in black soldier fly larvae impacts their growth and development. The excretion of Cr and Cd by black soldier fly larvae, especially the combined effects of Cr and Cd on larval growth and metal excretion, has rarely been studied. Given the prevalence of Cr and Cd in Chinese livestock and poultry manures (23), they were selected as the target heavy metals to study their combined exposure in BSFL, thereby extending existing knowledge beyond single-metal studies.

The gut microbiota plays a crucial role in the host’s ability to maintain health, immunity, metabolism, and nutrient absorption (24). The gut microbiota is influenced by environmental contaminants, and the patterns of these changes can differ significantly based on the types of pollutants and the host species involved (25, 26). Research has demonstrated the effects of heavy metals on black soldier fly larval development; however, little is known about the impact of heavy metals on the gut microbiota of black soldier fly larvae (22), particularly regarding the combined effects of multiple heavy metals. The response of the gut microbiota of black soldier fly larvae to high concentrations and combinations of Cr and Cd remains unclear. Clarifying this knowledge gap—understanding BSFL’s heavy metal tolerance and gut microbiota responses to Cr and Cd—will help establish standards for safe BSFL-derived livestock feed (to avoid heavy metal transfer through the food chain) and optimize BSFL-based manure bioremediation (to enhance Cr and Cd removal and reduce environmental risks), thereby bridging academic research and practical application.

The aims of this research are (1) to investigate the patterns of Cr and Cd accumulation in black soldier fly larval bodies and frass; (2) to study the impact of the heavy metals Cr and Cd, individually and in combination, on black soldier fly larval growth and development; and (3) to explore gut damage and microbiota of black soldier fly larvae under heavy metal stress caused by Cr and Cd, individually and in combination.

## Materials and methods

### Experimental materials and design

Eggs of black soldier fly (BSF) were purchased from a company (Wuliang Biotechnical Co., Ltd., Guangzhou, China). Egg hatching followed the method described by Hao et al. (27). Briefly, BSF eggs were incubated with wheat bran in a plastic box with a diameter of 10 cm and a height of 20 cm. Hatching conditions were a temperature of 28 °C and 75% humidity. The larvae were reared in wheat bran for 4 days following egg hatching. For larval exposure experiments, wheat bran was used as the rearing substrate and spiked with chromium or cadmium stock solutions. Chromium (Cr) and cadmium (Cd) stock solutions (1,000 mg L^−1^) were prepared using potassium dichromate (K_2_Cr_2_O_7_) and cadmium sulfate (CdSO_4_), respectively. For the single-metal treatments, wheat bran was mixed with appropriate volumes of Cr stock solution to achieve final concentrations of 100 and 600 mg Cr per kg dry wheat bran, designated as Cr100 and Cr600, respectively. Correspondingly, the Cd stock solution was added to wheat bran to obtain final concentrations of 20 and 120 mg Cd per kg dry wheat bran, designated as Cd20 and Cd120, respectively. Binary combined metal treatments were also established: Cr100Cd20, Cr100Cd120, Cr600Cd20, and Cr600Cd120. The exposure concentrations of Cr and Cd in this study were within the lowest and highest metal concentrations that have been reported for animal feed or contaminated manure. The highest value of Cr is 622.5 mg kg^−1^ in animal feed. The lowest value of Cd is 31.0 mg kg^−1^ in animal feed, and the highest value is 129.8 mg kg^−1^ in swine manure (20, 28, 29). Nine treatments were included, including control (CK), Cr100, Cr600, Cd20, Cd120, Cr100Cd20, Cr100Cd120, Cr600Cd20, and Cr600Cd120. Five replicates of each treatment were performed, and 2.0 g of four-day-old larvae were weighed and placed in each round plastic container (78 cm^2^ × 10 cm), wheat bran spiked with heavy metals (40 containers), and wheat bran spiked without heavy metals (5 containers). When the larvae reached the third instar stage (15 days), larvae and residue were manually separated.

### Larval growth and behavior

Every 2 days, thirty larvae were randomly chosen, weighed, and measured for length for each treatment. After that, the larvae were returned to the corresponding container.

Before subsequent operations (e.g., larval weight measurement, gut dissection, heavy metal detection), residual meal particles could adhere to larval surfaces, interfering with detection accuracy or tissue integrity. Therefore, isotonic phosphate-buffered saline (PBS) was used to wash larvae, as it removes surface particles without damaging them. The larvae were then allowed to crawl in the center of the agar plates (30). The agar plates were 90 mm in diameter and 10 mm in height, and 0.8% agar was used for stable crawling. The larval trailing path was recorded on the agar plate for 1 min. The traveling speed of the larvae in 1 min was calculated from their path and plotted. To avoid larval interactions, one larva per agar plate was used. For all measurements, the temperature was maintained at 25 ± 1 °C, and they were performed under indoor light.

### Larval crude protein and ether extract

The larvae were freeze-dried, mashed in a mortar, sieved through a 60-mesh sieve, and stored at −20 °C until measurement. Crude protein (CP) and ether extract (EE) in the insects were determined in accordance with the Chinese national standards GB/T 6432-2018 and GB/T 6433-2006, respectively.

### Trypan blue staining

Trypan blue staining is an established dye-exclusion assay used to assess cell viability (31). Due to the barrier function of intact cell membranes, trypan blue cannot enter viable cells, resulting in an unstained appearance (32). Consequently, Trypan blue staining was used to determine whether heavy metal stress caused gut damage. To this end, third instar larvae from each treatment were stained for 30 min with 0.2 mg/mL^−1^ of Trypan blue (33, 34). The stained larval bodies were imaged before dissection, and the guts were imaged after dissection using a stereo microscope (NOVEL, Nanjing, China), and the images were processed to determine whether cell damage was present.

### Heavy metal analysis

The larval bodies were dried to constant weight at 105 °C in a forced-air drying oven (DHG-9070A, Shanghai Jinghong, China), and the larval feces were dried in air and sifted through a 1 mm mesh for heavy metal analysis. Larval feces and bodies were digested for 25 min in a 10 mL solution of HNO_3_ and H_2_O_2_ (4,1, v/v) using microwave digestion equipment (TANK 40, Sineo, Shanghai, China) with the temperature ramped to 120 °C at 5 °C/min (held 5 min), then to 180 °C at 8 °C/min (held 15 min). The maximum pressure was 2.0 MPa and the power 1,200 W. Inductively coupled plasma mass spectrometry was used to determine Cr and Cd concentrations (ICP–MS, iCAPQ, Thermo Scientific, Bremen, Germany).

### Gut bacterial community analysis

The larval samples from nine treatment groups (five replicates each) were analyzed using high-throughput sequencing by Illumina NovaSeq 6,000 (Illumina, Santiago, CA, United States). The DNA was extracted with the TGuide S96 Magnetic Soil /Stool DNA Kit (Tiangen Biotech (Beijing) Co., Ltd.) according to the manufacturer’s instructions. The DNA concentration of the samples was measured with the Qubit dsDNA HS Assay Kit and Qubit 4.0 Fluorometer (Invitrogen, Thermo Fisher Scientific, Oregon, United States). The 338F: 5’-ACTCCTACGGGAGGCAGCA-3′ and 806R: 5′- GGACTACHVGGGTWTCTAAT-3′ universal primer set was used to amplify the V3–V4 region of the 16S rRNA gene from the genomic DNA extracted from each gut DNA sample (35). The constructed library was sequenced on an Illumina NovaSeq 6,000 (Illumina, Santiago, CA, United States) for sequencing. Based on single-nucleotide quality scores, raw data were primarily filtered by Trimmomatic (36) (version 0.33). Identification and removal of primer sequences were performed by Cutadapt (37) (version 1.9.1). PE reads obtained from the previous steps were assembled by USEARCH (38) (version 10) and followed by chimera removal using UCHIME (39) (version 8.1). The high-quality reads generated from the above steps were used for downstream analysis. Sequences with similarity ≥ 97% were clustered into operational taxonomic units (OTUs) by USEARCH (36) (version 10.0), and the OTUs with abundance < 0.005% were filtered. Taxonomic annotation of the OTUs was performed based on the Naive Bayes classifier in QIIME2 (40) using the SILVA database (39) (release 132) with a confidence threshold of 70%.

### Statistical analysis

**Figure 1:**
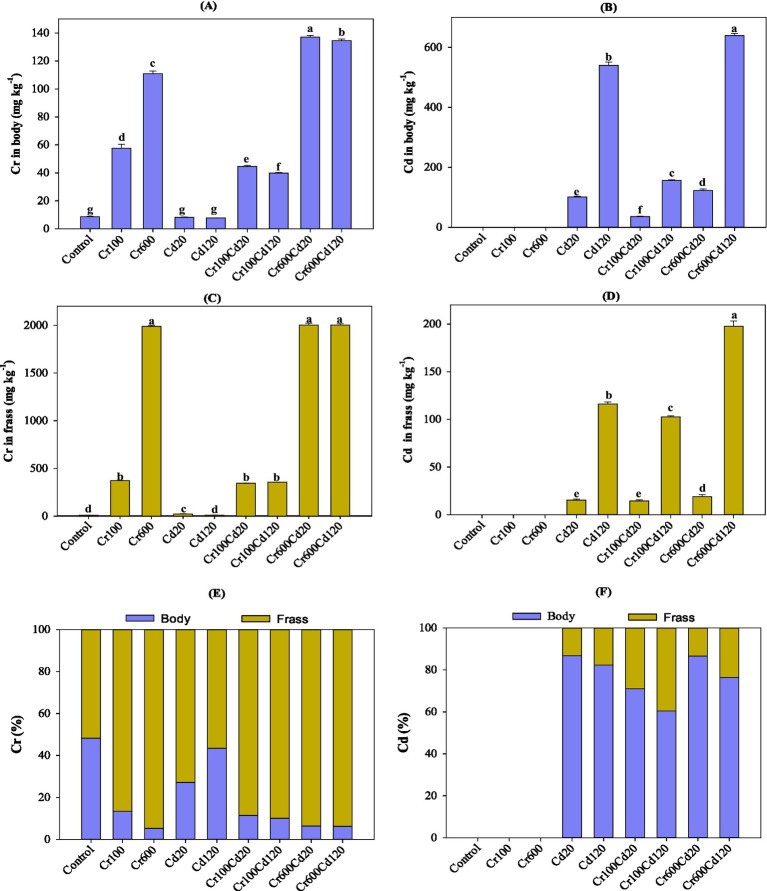
Cr **(A)** and Cd **(B)** accumulation in the larval body; Cr **(C)** and Cd **(D)** excretion in the frass; Cr **(E)** and Cd **(F)** distribution in the larval bodies and frass. Error bars represent the standard deviation of five replicate measurements (*n* = 5). Columns marked by the same lowercase letter indicate that the values are not significantly different (*p* > 0.05).

**Figure 2:**
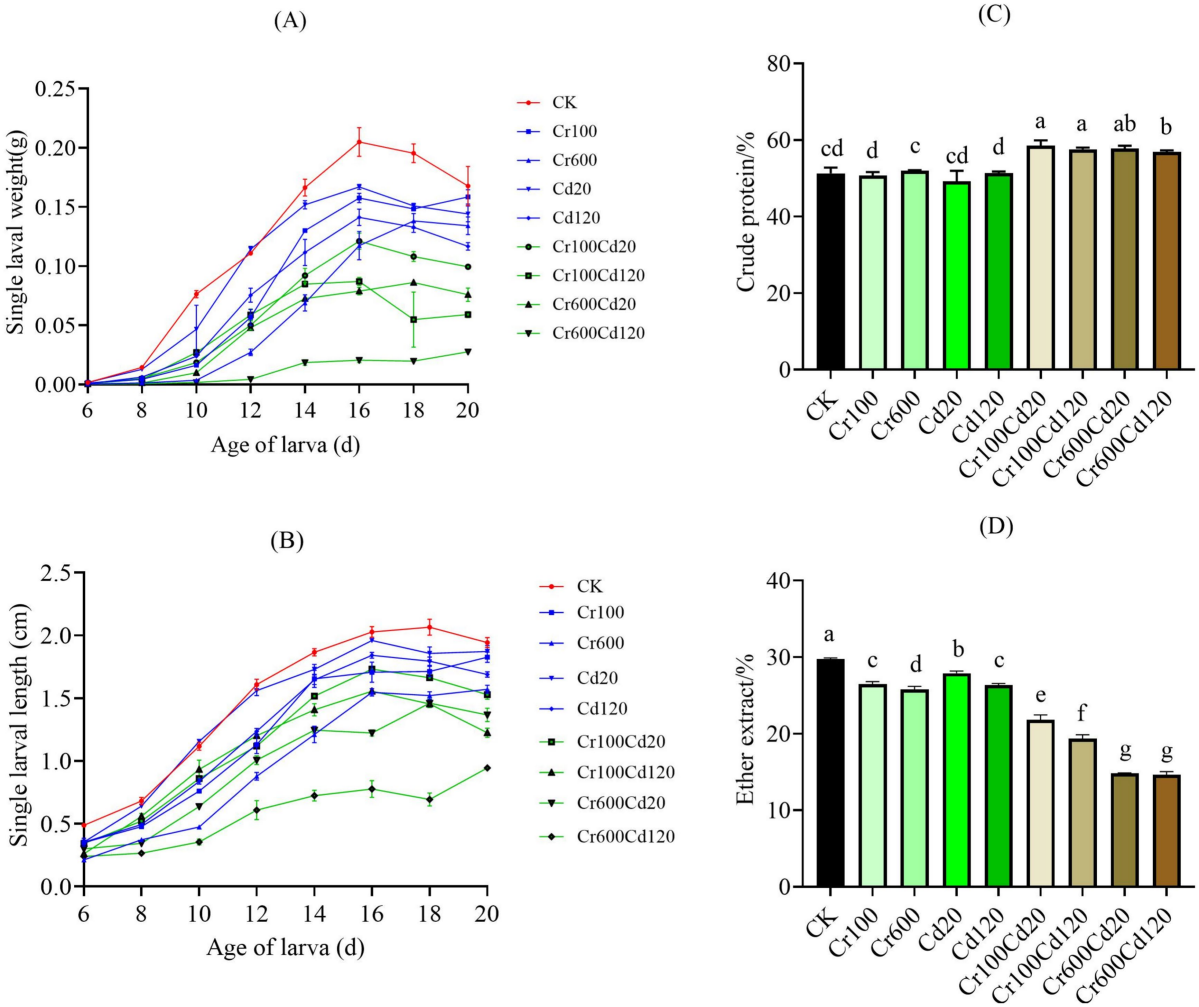
Larval weight **(A)**, length **(B)**, crude protein content **(C)**, and ether extract content **(D)** under different treatments. Error bars represent the standard deviation of five replicate measurements (*n* = 5). Columns marked by the same lowercase letter are not significantly different (*p* > 0.05).

**Figure 3:**
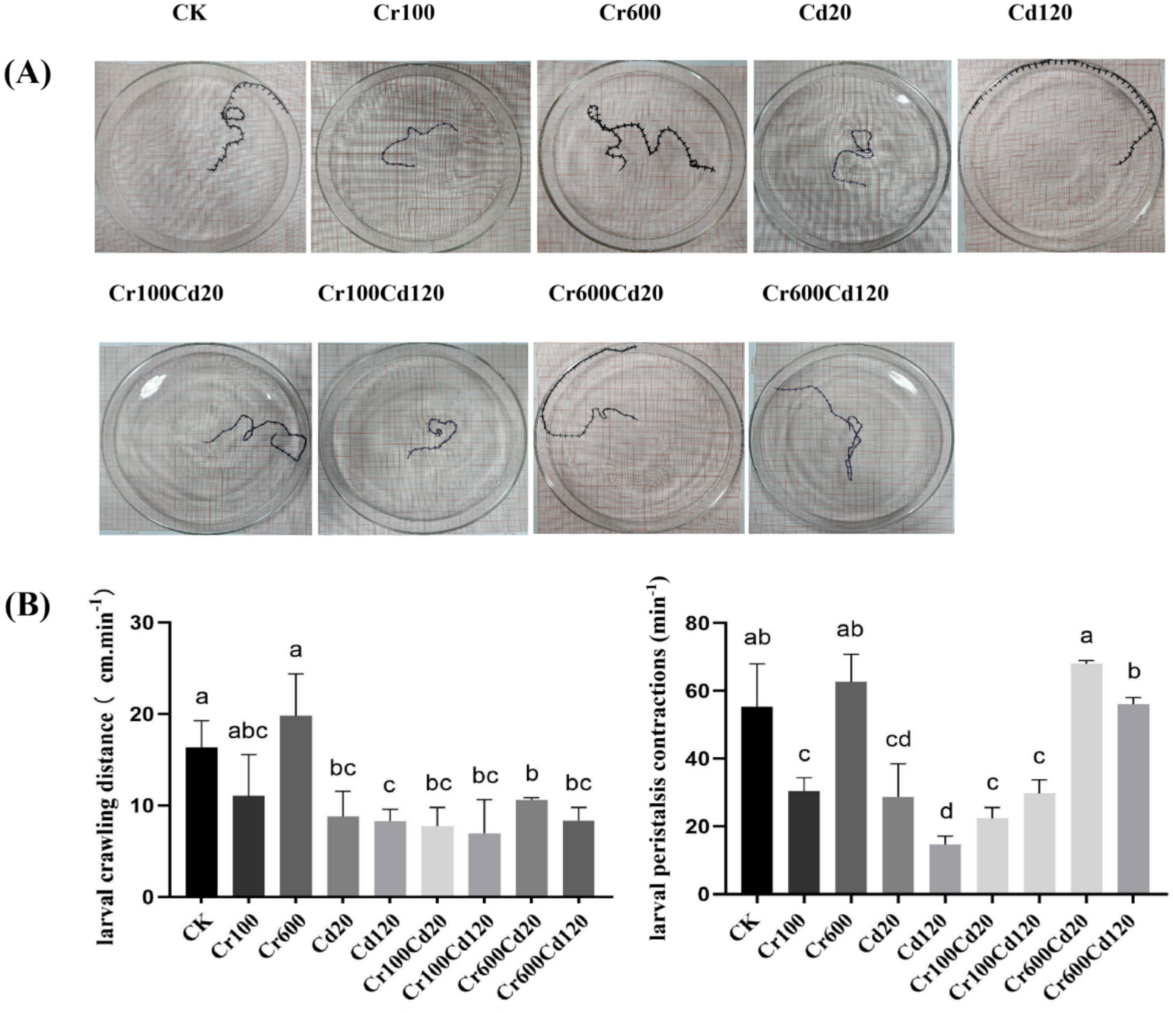
Photograph of third-instar larval crawling route **(A)**; statistics of larval crawling speed and larval peristaltic contraction frequency **(B)** under different treatments. Error bars represent the standard deviation of three replicate measurements. Columns marked by the same lowercase letter are not significantly different (*p* > 0.05).

**Figure 4:**
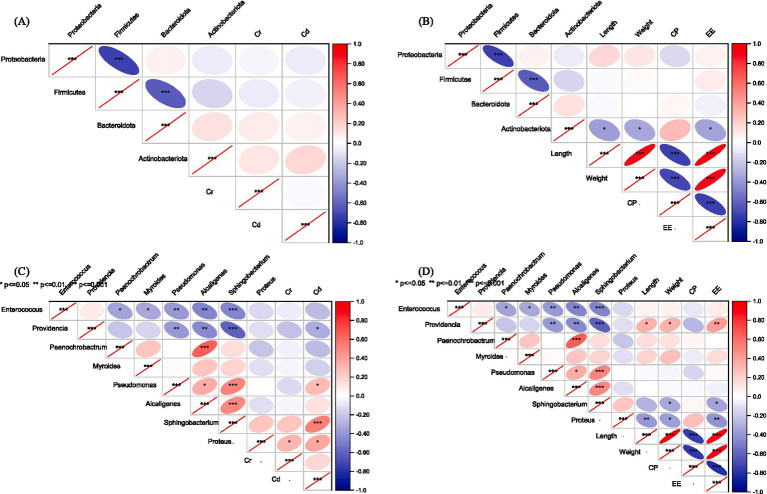
Spearman correlation analysis of the abundances of the main larval gut bacterial phyla **(A)** and genera **(C)** with Cr and Cd in the larval body across all groups and the abundances of the main larval gut bacterial phyla **(B)** and genera **(D)** and larval length, weight, CP, and EE across all groups. Red and blue colors indicate positive and negative correlations. ^*^ *p* < 0.05, ^**^ *p* < 0.01, and ^***^ *p* < 0.001.

The results of a one-way analysis of variance (ANOVA) and the least significant difference (LSD) multiple comparison test to determine statistically significant differences between treatments were conducted and analyzed using the Statistical Package for Social Sciences (SPSS version 25.0). The abundance of bacterial communities was illustrated using the R software (version 3.6.1). The online drawing tool LEfSe^1^ was used to produce UpSet plots to analyze the distinct taxonomic features of the treatments. The alpha-diversity indices (Chao1 and Shannon) were calculated and analyzed using QIIME2 and R software (version 3.4.3) with the vegan package. Beta diversity was determined to evaluate the similarity among microbial communities from different samples using QIIME2. Nonmetric multidimensional scaling (NMDS) was used to analyze the beta diversity. The relationship between bacterial taxa and heavy metal concentrations was analyzed by Pearson correlation. Gephi software was used to perform microbial co-occurrence network analysis, and other plots in Figures 1–4 were generated using Origin 2021 software.

## Results

### Cr and Cd accumulation and distribution in the larval body and frass

Cr and Cd in the diet were transferred into larval bodies, where both accumulated and were significantly higher at Cr600 and Cd120 treatments than at Cr100 and Cd20 treatments, respectively. The Cr content of the larval bodies significantly (*p < 0.05,* least significant difference, LSD test) increased about 6.7- and 12.9-fold for Cr100 and Cr600 treatments, respectively, compared with the control (CK) (8.6 mg kg^−1^) treatment (Figure 1A). Similarly, the amount of Cd in the larval bodies significantly (*p < 0.05,* LSD test) increased 101.1- and 539.9-fold for Cd20 and Cd120 treatments, respectively, compared with the CK treatment (Figure 1B). Moreover, the combined Cr–Cd treatments showed that simultaneous exposure to high levels of Cr, at 600 mg kg^−1^, and Cd, at 120 mg kg^−1^, resulted in correspondingly higher heavy metal accumulation in the larval body (Figures 1A,D). The contents of Cr and Cd in larval frass showed a similar trend to those in larval bodies (Figures 1C,D).

The cumulative percentages of Cr (86.6–94.7%) in the frass were clearly higher than those in the bodies of Cr-exposure groups (5.3–13.4%), but the cumulative percentages of Cd (13.2–39.6%) in the frass were clearly lower than those in the bodies of Cd-exposure groups (60.4–86.8%) (Figures 1E,F). In brief, as the exposure concentration was increased, the larval bodies accumulated both Cr and Cd, and their contents in frass significantly increased. Notably, Cd accumulated in larval bodies more readily than Cr at the tested concentrations. The accumulation proportions were 60.4–86.8% for Cd and <15% for Cr, relative to the total heavy metals in both the bodies and frass.

### Heavy metals’ influence on larval development

The larvae in the CK group developed rapidly, and the larval body weight showed significant differences among all groups (F _8, 63_ = 2.500; *p* = 0.0200, one-way ANOVA). The CK had the highest body weight among heavy metal-treated groups (Figure 2A). Although there was no significant treatment effect on larval body length among all groups (F _8, 63_ = 1.970; *p* = 0.0649, one-way ANOVA), the CK exhibited the highest body length among most of the experimental groups (Figure 2B). The lowest larval body weight and length were found in the Cr100Cd120 group. Larval weight and length were lower after feeding with diets with Cr and Cd, especially for combined Cr and Cd groups, compared with the single Cr or Cd and CK (Figure 2).

There was a significant effect (F_8, 36_ = 43.59; *p* < 0.0001, one-way ANOVA) between the groups in larval crude protein (CP) contents (Figure 2C). The crude protein content of the larvae in the combined Cr and Cd groups was significantly higher than in other groups (*p* < 0.05, LSD test). The crude protein content did not differ significantly among the CK, Cr100, Cr600, and Cd120 groups. A significant impact was also found for ether extract (EE) (F_8,36_ = 1,103; *p* < 0.0001, one-way ANOVA). The CK group had significantly higher EE in larvae than the other Cr and/or Cd-added groups (*p* < 0.05, LSD test). EE values decreased significantly (*p* < 0.05, LSD test) with increasing Cr and Cd concentrations. Combined Cr–Cd treatments showed significantly (*p* < 0.05, LSD test) lower EE values than the single Cr and Cd treatments. Furthermore, high combined concentration groups (Cr600Cd120 and Cr100Cd120) exhibited lower EE contents than low combined concentration groups (Cr600Cd20 and Cr100Cd20) (Figure 2D).

### Heavy metals’ influence on larval behavior and gut damage

The crawling speed of the third-instar larvae showed that larvae in the CK and Cr600 groups can cover more distance than the other groups (Figure 3A). The crawling speed showed significant differences among all groups (F _8, 18_ = 6.454; *p* = 0.0005, one-way ANOVA). Generally, crawling speed decreased with the addition of heavy metal (Figure 3B). The larval peristalsis showed significant differences among groups (F _8, 18_ = 27.34; *p* < 0.0001, one-way ANOVA). The CK and Cr-added 600 mg kg^−1^ groups (Cr600, Cr600Cd20, and Cr600Cd120) had significantly (*p <* 0.05, LSD test) higher peristalsis than the other groups (Figure 3B).

**Figure 5:**
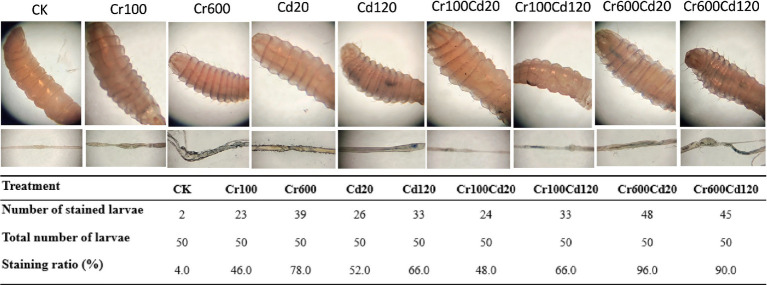
Photographs of trypan blue staining and the ratio of third instar larvae from different treatments.

*In vitro* and dissected gut images of larvae from different treatments following trypan blue staining are presented in Figure 5. Larvae were randomly chosen from groups treated with different concentrations of Cr and Cd and exhibited varying degrees of staining. Statistical analysis demonstrated that approximately 4.0% of larvae in the CK group displayed stained midguts, whereas the proportion of stained larvae in the other groups ranged from 46.0 to 96.0% (Figure 5). Larvae remained viable throughout the staining procedure until photographic documentation and *in vitro* observation.

### Influence on larval gut bacterial community

**Figure 6:**
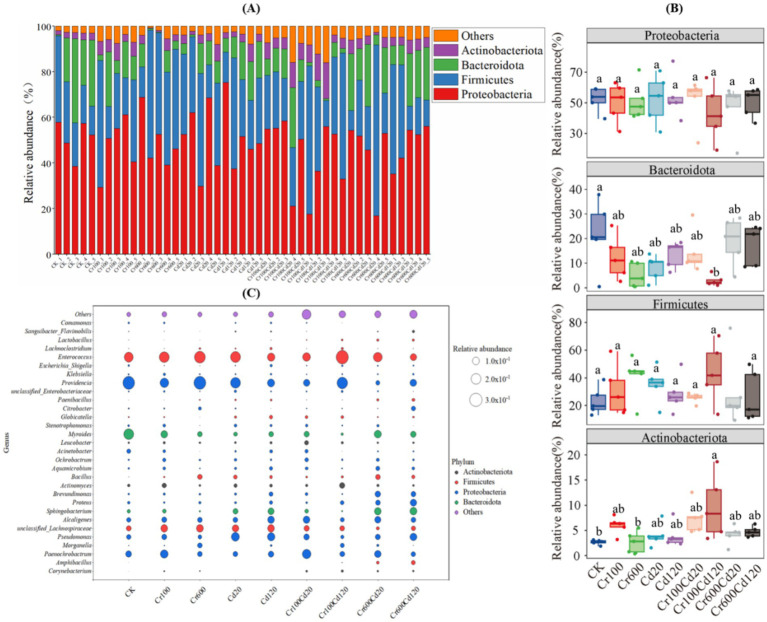
Larval gut bacterial composition at the phylum level **(A)**, statistical analysis of four phyla **(B)**, and genus level **(C)** (mean, *n* = 5) for different treatments. Columns marked by the same lowercase letter are not significantly different (*p* > 0.05). Bacterial OTUs accounting for less than 1% of the total number of reads are categorized as “others”.

The dominant phyla were Proteobacteria (50.06%), Firmicutes (30.52%), Bacteroidetes (12.70%), and Actinobacteria (average 5.05%) (Figure 6A). There were no statistical differences (*p* > 0.05, LSD test) among treatments in the relative abundance of Proteobacteria and Firmicutes. The relative abundance of Bacteroidetes showed no significant difference among treatments (*p* > 0.05, LSD test), except that the CK group was significantly higher than the Cr100Cd120 group (*p* < 0.05, LSD test). No significant difference in the relative abundance of Actinobacteria was observed among treatments (*p* > 0.05, LSD test), except that the CK and Cr600 groups were significantly lower than the Cr100Cd120 group (*p* < 0.05, LSD test) (Figure 6B). Moreover, there were 29 genera that had relative abundances >1%. *Enterococcus* and *Providencia* were the most abundant genera (average 16.93 and 15.63%, respectively) (Figure 6C; Supplementary Table S1).

**Figure 7:**
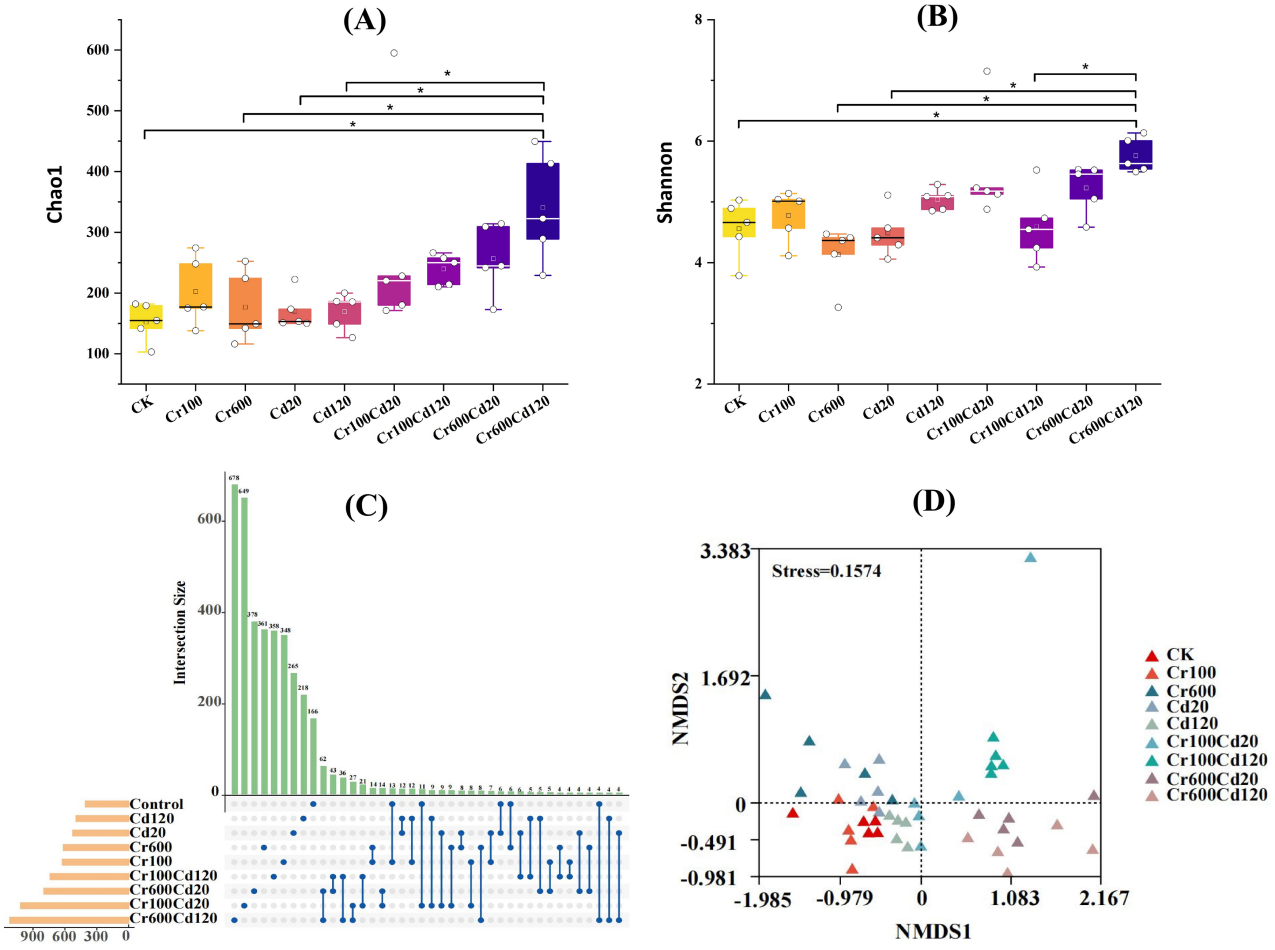
Larval gut bacterial community diversity indices (**A**: Chao1; **B**: Shannon). UpSet plots display the number of bacterial OTUs in the larval gut for different groups **(C)**. NMDS analysis based on the Bray–Curtis distance **(D)**.

**Figure 8:**
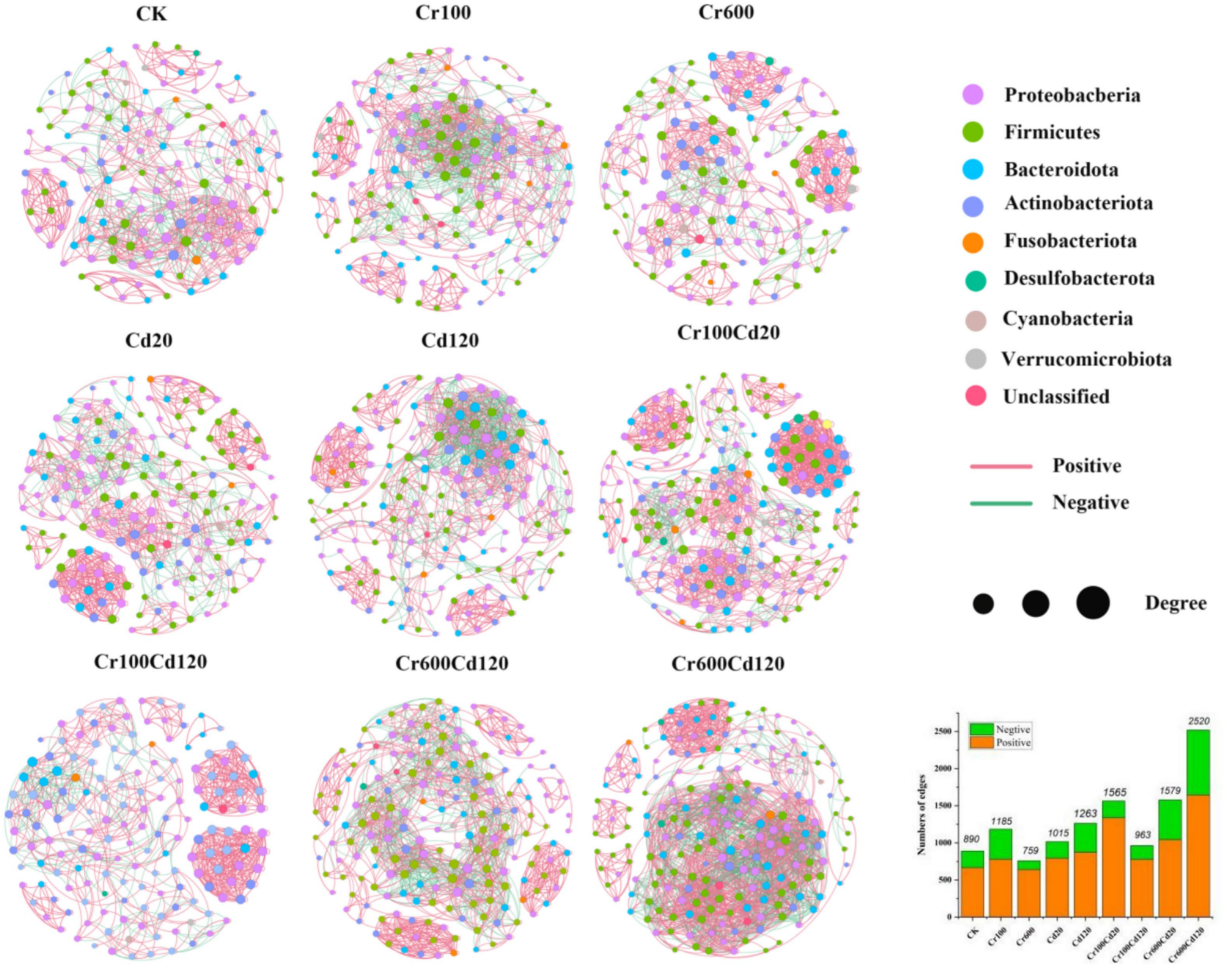
Gut microbial networks. Nodes are colored according to phylum level, and node size denotes the number of connections.

The Chao1 diversity index for the high exposure Cr–Cd group (Cr600Cd120) was significantly (*p* < 0.0*5,* LSD test) higher than that of Cr600, Cd20, Cd120, and CK treatments (Figure 7A). The Shannon diversity index for the Cr600Cd120 treatment was significantly (*p <* 0.05, LSD test) higher than that of the Cr600, Cd20, Cr100Cd120, and CK treatments (Figure 7B). Changes in gut community diversity were further supported by the UpSet plot analysis, which revealed that higher numbers of unique OTUs in Cr, Cd, and combined Cr–Cd than in the CK group (Figure 7C). Cr, Cd, and combined Cr–Cd treatments at the tested exposure concentrations significantly altered the bacterial community structure of the larval gut, with a stress value of 0.1574 (Figure 7D). The complexity of the bacterial community networks was generally higher in Cr- and Cd-added groups (963–2,520) than in the CK group (890), except for Cr600 (Figure 8).

### Correlation of heavy metals in the larval body with gut bacteria

The relative abundances of Proteobacteria and Firmicutes were negatively correlated with Cr and Cd concentrations in larval bodies, whereas those of Bacteroidetes and Actinobacteria were positively correlated (Figure 4A). Accordingly, the Proteobacteria and Firmicutes showed positive correlations, whereas Bacteroidetes and Actinobacteria showed negative correlations, with larval weight and EE (Figure 4B). *Enterococcus*, *Providencia*, *Paenochrobactrum,* and *Myroides* showed negative correlations with Cr and Cd and were positively correlated with larval length, weight, and EE. *Pseudomonas*, *Alcaligenes*, *Sphingobacterium,* and *Proteus* showed positive correlations with Cd and were negatively correlated with larval length, weight, and EE (Figures 4C,D; Supplementary Table S1).

## Discussion

The accumulation and excretion of Cr and Cd in the larval bodies and frass increased with increasing heavy metal concentrations, which is consistent with previous studies (17, 22). Moreover, the cumulative percentages of Cd (60.4–86.8%) in larval bodies were higher in Cd-exposure groups than the cumulative percentages of Cr (5.3–13.4%) in Cr-exposure groups (Figures 2C,F), indicating that Cd is more likely to accumulate in larval bodies than Cr. This finding is consistent with a previous study by Gao et al. (20), which found that Cd tends to accumulate at high levels in black soldier fly larval bodies. These variations suggest that heavy metal distribution between frass and insect bodies is strongly associated with metal type and concentration (16, 21, 22). The high accumulation of Cd may be attributed to Cd^2+^ being easily absorbed by cells through Ca^2+^ channels, as it is similar to Ca^2+^ (17, 41). Heavy metals accumulate in insects and impact their growth, development, and physiological function (42). Insects have developed detoxification mechanisms to reduce heavy metal toxicity and maintain internal homeostasis, including excretion via frass (43). In this study, Cr primarily accumulated and was distributed in the frass of black soldier fly larvae, whereas Cd primarily accumulated in larval bodies. This difference may be attributed to the fact that Cd^2+^, with an ionic radius and physicochemical properties similar to Ca^2+^, can easily enter cells via Ca^2+^ channels. Once absorbed, Cd is poorly excreted and thus highly bioaccumulated in larvae’s bodies. In contrast, Cr mainly exists as anionic complexes (e.g., CrO_4_^2−^) under physiological conditions, which cannot be efficiently taken up by cells and are rapidly eliminated in the frass, leading to low accumulation in larvae (17, 41).

The larval growth decreased in response to single Cr exposure at concentrations of 100 and 600 mg kg^−1^, and single Cd exposure at 20 and 120 mg kg^−1^ (Figures 2A,B). A similar trend was also observed by Wu et al. (22), who reported that larval weight was the highest when exposed to Cd20, but no significant differences were observed among the Cd treatments and the CK group. Gao et al. (20) found that Cd had no effect on larval survival and eclosion rate when the concentration of Cd was 80 mg kg^−1^. In our study, we found that larval weight and length decreased after feeding with diets containing Cr and Cd, especially in the combined Cr and Cd groups, which significantly inhibited larval growth. This finding is consistent with other studies showing that heavy metal concentrations above a certain threshold inhibit weight gain (44, 45). However, no larval mortality was observed during the experimental period. Based on these results, black soldier fly larvae appear to tolerate exposure to Cr and Cd at the tested levels. Similarly, Cai et al. (19) also reported that black soldier fly larvae exhibited high tolerance to diets containing mixed heavy metals.

In another aspect, larval growth was inhibited by the heavy metals Cd and Cr, as the ether extract (EE) was significantly affected. Specifically, significantly lower EE was found in the Cr and/or Cd-exposure groups than in the CK group (Figure 2D). With increasing Cr and Cd concentrations, EE decreased significantly, and the combined Cr and Cd groups had significantly lower EE than the single Cr and Cd groups; this difference may be attributed to synergistic toxicity that disrupts lipid metabolism and gut microbiota. Specifically, lipid synthesis inhibition may be a key contributor; Cr suppresses enzymes, Cd induces ROS-mediated damage, and their combination exacerbates this blockage to further reduce EE. Gut microbiota dysbiosis may further aggravate EE reduction; combined Cr/Cd exposure more severely reduced *Enterococcus* compared to single-metal groups, thereby impairing lipid utilization. Notably, larval crude protein (CP) content in the combined Cr and Cd groups was significantly higher than that in other groups, and there were no significant differences among the CK, Cr100, Cr600, and Cd120 groups (Figure 2C). The possible reason for the high CP content in the larvae is that heavy metals trigger an oxidative stress response, leading to the synthesis of a series of stress proteins (46, 47), such as heat shock proteins. Increased synthesis of these proteins may improve the larvae’s ability to survive under adverse conditions, resulting in higher total protein levels. Moreover, heavy metal stress may alter metabolic pathways in larvae. For example, in response to heavy metal toxicity, larvae may enhance certain metabolic processes, such as detoxification metabolism (48, 49). This may involve the synthesis of additional enzymatic proteins to participate in the detoxification reaction, resulting in a rise in body protein content (50). In addition, heavy metal stress usually puts additional physiological stress on larvae, causing them to require more energy to survive and cope with the stress. Protein is one of the important energy sources (51). Larvae may need to continue to grow and undergo tissue repair despite being under heavy metal stress, and proteins are important components of cell growth and repair (52). Thus, in order to meet the increased energy, growth, and repair demands, larvae may synthesize more proteins to support these physiological processes.

Heavy metals Cr and Cd pose distinct toxic risks to Chironomus kiiensis larvae, *Apis cerana*, and black soldier fly larvae (BSFL). In general, Cd shows higher acute toxicity and genotoxicity than Cr across three species (49, 53, 54). Chironomus kiiensis larvae are the most sensitive, exhibiting severe DNA damage and developmental abnormalities even at low Cd concentrations (53). *Apis cerana* exhibits decreased survival and impaired immune function under metal exposure (55). In contrast, BSFL display strong tolerance to heavy metals, with toxicity mainly manifested as growth inhibition and gut microbiota disturbance, which makes them promising candidates for heavy metal bioremediation (49, 56). This study demonstrates for the first time that we report that Cr and Cd can affect the mechanosensory organs and behavior of the black soldier fly larvae. The behavioral defect is often associated with the functionality of the neurons (57). Motor neurons in the larval brain control muscle contraction required for crawling and moving the fruit fly (57). Black soldier fly larvae’s climbing activity is also one of the indicators of neuronal homeostasis (58). In the present study, crawling speed generally decreased with increasing heavy metal concentrations. This reduction in locomotor activity may reflect the neurotoxic effects induced by Cd and Cr. Heavy metals can impair neuronal function by interfering with nerve growth factors, voltage-gated channels in hippocampal neurons, and the integrity of the blood–brain barrier (BBB), ultimately leading to neurodegeneration (59). Larval crawling behavior is a widely used assay to evaluate neuronal function, as climbing represents a coordinated motor activity involving multiple muscles and neurons (60). Thus, during locomotion, the central nervous system, the peripheral nervous system, and muscles are involved. Larval crawling is regulated by a central pattern generator (CPG) to produce rhythmic motion in larvae (61).

The dominant phyla identified in this study were Proteobacteria, Firmicutes, Bacteroidetes, and Actinobacteria. Similar findings regarding the predominant gut microbiota of black soldier fly larvae have been reported in previous studies (14, 22, 62). Moreover, *Enterococcus* and *Providencia* were the most abundant genera in this study, which aligns with the findings of Hammer et al. (63), who noted that *Enterococcus* is a dominant genus in the gut of black soldier fly larvae and exhibits certain tolerance to heavy metals. *Enterococcus* has been widely reported to participate in nutrient metabolism, including the breakdown and absorption of organic matter, which may directly promote larval development. In addition, some members of *Enterococcus* possess metal resistance mechanisms and may alleviate heavy metal toxicity via biosorption or detoxification, thereby protecting the host from oxidative damage and maintaining normal physiological function. The NMDS analysis showed that Cr, Cd, and the combination of Cr and Cd at the tested exposure concentrations significantly altered the bacterial community structure of the black soldier fly larvae gut. This may reflect that Cr and Cd, as factors in the external environment, can influence the gut microbial flora of black soldier fly larvae to some extent (63). For example, Proteobacteria and Firmicutes showed negative correlations, while Bacteroidetes and Actinobacteria showed positive correlations with Cr and Cd concentrations in larval bodies. In contrast, Proteobacteria and Firmicutes showed positive correlations, while Bacteroidetes and Actinobacteria showed negative correlations with larval weight and EE (Figure 4). These results suggest that Cr and Cd accumulated in larval bodies could affect larval growth and development by altering their gut microbial flora.

The changes in gut community structure and diversity are further supported by the UpSet plot, which showed that the Cr/Cd combination groups generally increased community diversity. This pattern may be associated with concentrations of Cr and Cd. However, this result differs from that reported in a previous study by Wu et al. (22), who found that exposure to high concentrations of single heavy metals reduced the gut bacterial diversity of black soldier fly larvae fed with pig manure. In contrast, our study showed an increase in diversity, which may reflect the combined exposure of Cr and Cd in our experiment and the complexity of the pig manure matrix, specifically its complex nutritional composition and physicochemical properties, which can affect the gut microbiota (22). The intricate relationships between microorganisms can be examined using co-occurrence networks, which provide the mathematical support for the aggregation of microbial communities. More nodes and links reflect greater network complexity. In a relatively healthy gut, cooperation may represent a key mode of interaction between bacteria (27, 64). Our findings suggest that exposure to Cr and Cd may lead to the formation of a more complex and cooperative microbial community in black soldier fly larvae.

It is well known that pollutants can influence animal growth and development by modulating the gut microbiota, which in turn affects host physiological processes such as nutrient digestion and absorption, lipid and glucose metabolism, and immune function (4, 26). In this study, exposure to Cr and Cd, particularly at high and combined concentrations (Cr600, Cr100Cd20, Cr600Cd20, and Cr600Cd120), significantly inhibited weight gain in black soldier fly larvae and altered the structure and diversity of their gut microbiota. We hypothesize that the gut microbiota may protect the host from contaminant-induced damage by rapidly enhancing host resistance to pollutants (22), for instance, by supplying essential nutrients, strengthening immune function, and outcompeting pathogenic or harmful microorganisms (4, 65, 66).

Notably, some studies have examined the effects of heavy metals on insect gut microbiota, with findings consistent with our observations. Specifically, Wang et al. (67) found that Cd exposure reduced gut microbiota diversity in Chilo suppressalis, altered the abundance of specific taxa, and associated functional pathways. Similarly, Yang et al. (68) showed that Cd exposure disrupted the gut microbial community of *Spodoptera frugiperda*, characterized by a decrease in the beneficial genus Enterococcus and an increase in heavy metal-resistant taxa (e.g., Pseudomonas). In addition, Varg et al. (69) demonstrated that Cr exposure induced gut dysbiosis and reduced microbial diversity in *Daphnia magna*. Collectively, these studies confirm that heavy metal exposure induces gut microbiota dysbiosis across diverse taxa, consistent with our findings in BSFL, and further support the universality of this phenomenon.

This study also has several limitations that should be acknowledged. First, wheat bran was used as the rearing substrate, which is simpler than real livestock manure (containing complex organic matter and other contaminants); this may limit the extrapolation of our findings on larval growth responses to Cr/Cd stress to practical livestock manure treatment conditions. Second, our focus was on the short-term effects of Cr and Cd exposure on larval development; long-term effects—such as changes in pupation rate and adult fitness—were not investigated.

## Conclusion

Larval biomass growth of black soldier fly (*Hermetia illucens*) was inhibited by exposure to 100 and 600 mg kg^−1^ of Cr and 20 and 120 mg kg^−1^ of Cd and their combined groups. Cr and Cd accumulation in *H. illucens* larvae and frass increased significantly with increasing exposure concentration. Notably, Cd accumulated more readily (60.4–86.8%) than Cr (<15%). Cr and Cd inhibited larval development, reduced larval weight, increased crude protein content, and decreased ether extract (EE) content. The dominant larval gut bacterial phyla were Proteobacteria, Firmicutes, Bacteroidetes, and Actinobacteria. Gut bacterial diversity and complexity generally increased with exposure; Proteobacteria/Firmicutes were positively correlated, while Bacteroidetes/Actinobacteria were negatively correlated with larval weight and EE. These findings suggest that Cr/Cd accumulation and excretion affect larval growth via gut microbiota alteration. Overall, black soldier fly larvae exhibited relatively high tolerance to Cr and Cd and their combination, even at high exposure concentrations.

## Data Availability

The data presented in this study are deposited in the National Center for Biotechnology Information (NCBI) Sequence Read Archive database (SRA) received the sequencing data, accession number is PRJNA1444492, the direct link is https://www.ncbi.nlm.nih.gov/sra/PRJNA1444492.

## Footnotes

1
http://huttenhower.sph.harvard.edu/lefse/
