# Supplementary material for: Influence of Cr and Cd accumulation and excretion on growth and gut microbiota in black soldier fly larvae (Diptera: Stratiomyidae)
Source: Front Vet Sci. 2026 Apr 15;13:1777366. doi: 10.3389/fvets.2026.1777366 (PMC13124520; doi:10.3389/fvets.2026.1777366)
Supplement: Supplementary file 1 [file Table_1.docx]

Table S1. The average relative abundance of larval gut bacterial composition at the genus level (mean, n = 5) for different treatments.
